# The placental tryptophan pathway across gestation: implications for pregnancy outcomes

**DOI:** 10.1093/humupd/dmag001

**Published:** 2026-02-19

**Authors:** Rona Karahoda, David Walker, Cilia Abad, Kasin Yadunandam Anandam, Padma Murthi, Frantisek Staud

**Affiliations:** Department of Pharmacology and Toxicology, Faculty of Pharmacy in Hradec Kralove, Charles University, Hradec Kralove, Czech Republic; The Ritchie Centre, The Hudson Institute of Medical Research, Monash University, Melbourne, VIC, Australia; Department of Pharmacology and Toxicology, Faculty of Pharmacy in Hradec Kralove, Charles University, Hradec Kralove, Czech Republic; Department of Pharmacology and Toxicology, Faculty of Pharmacy in Hradec Kralove, Charles University, Hradec Kralove, Czech Republic; Department of Maternal Fetal Medicine, Pregnancy Research Centre, Royal Women’s Hospital and First Year College and Institute of Health and Sport, Victoria University, Melbourne, VIC, Australia; Department of Pharmacology and Toxicology, Faculty of Pharmacy in Hradec Kralove, Charles University, Hradec Kralove, Czech Republic

**Keywords:** placenta, tryptophan metabolism, fetal programming, neurodevelopment, DOHaD, serotonin, melatonin, kynurenine

## Abstract

**BACKGROUND:**

Tryptophan metabolism within the placenta generates bioactive metabolites, including serotonin (5-hydroxytryptamine; 5-HT), melatonin, and kynurenine derivatives, that regulate immune tolerance, vascular function, oxidative balance, and fetal neurodevelopment. Increasing evidence indicates that placental handling of tryptophan is dynamically regulated across gestation and is highly sensitive to maternal environmental and metabolic cues.

**OBJECTIVE AND RATIONALE:**

The aim of this review is to examine placental tryptophan metabolism across gestation, with a focus on the 5-HT, melatonin, and kynurenine pathways. We address how these pathways are regulated during normal pregnancy and how maternal factors, including inflammation, hypoxia, oxidative stress, and cardiometabolic dysfunction, influence placental tryptophan handling in pregnancy complications such as early pregnancy loss, preeclampsia, fetal growth restriction, and preterm birth.

**SEARCH METHODS:**

PubMed was searched using predefined terms related to placental tryptophan metabolism, 5-HT, melatonin, kynurenine, fetal programming, neurodevelopment, and pregnancy complications. Only full-text, peer-reviewed articles published in English were included. Abstracts and conference proceedings were excluded due to their limited data reliability.

**OUTCOMES:**

Placental tryptophan metabolism shows clear gestational stage-dependent regulation, and early pregnancy emerges as a formative period when pathway activity and metabolite balance are first established. From early pregnancy, maternal–decidual kynurenine pathway activity and placental 5-HT synthesis intersect with immune tolerance, vascular adaptation, and neurodevelopmental signaling. Across gestation, maternal inflammation, hypoxia, oxidative stress, and cardiometabolic disturbance can redirect the tryptophan flux and shift the balance between 5-HT/melatonin and downstream kynurenine metabolites. Evidence across pregnancy complications links early pathway disruption to pregnancy loss and supports the view that early metabolic perturbations contribute to vulnerability for later placental dysfunction, including preeclampsia, fetal growth restriction, and preterm birth.

**WIDER IMPLICATIONS:**

Placental tryptophan metabolism changes across gestation, making early pregnancy a critical window when pathway balance and fetal exposure to neuroactive metabolites are first set. Maternal inflammation, metabolic status, nutrition, and drug exposures may alter this balance, with the placenta acting as the key interface that transmits maternal signals to the fetus and shapes neurodevelopmental trajectories. To define the clinical relevance of altered tryptophan catabolism, longitudinal human studies are needed to link placental phenotypes with pregnancy outcomes and postnatal neurodevelopment. These should be complemented by mechanistic models that resolve regulation in early gestation.

**REGISTRATION NUMBER:**

n/a.

## Graphical abstract

**Figure:**
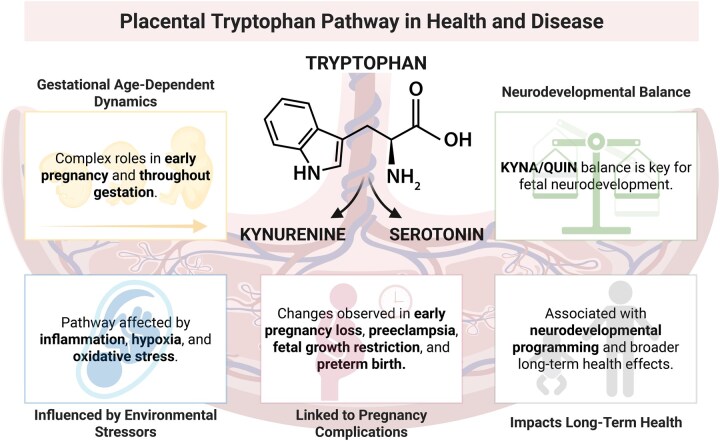
**Tryptophan metabolism in the placenta is dynamically regulated across gestation and contributes to fetal neurodevelopment, pregnancy complications, and long-term offspring health.** KYNA, (neuroprotective) kynurenic acid; QUIN, (neurotoxic) quinolinic acid. Created in BioRender https://BioRender.com/xh0t3fh.

## Introduction

The placenta is a central regulator of the maternal–fetal interface, coordinating nutrient exchange, hormonal signaling, and immune adaptation throughout pregnancy (Knofler *et al*., 2019). In addition to supporting fetal growth, placental function is highly responsive to maternal physiological and environmental conditions, including inflammation, stress, metabolic status, genetic variation, and exposure to xenobiotics (Jansson and Powell, 2007). While this adaptability generally promotes fetal survival, it can also induce persistent changes in fetal physiology that increase susceptibility to metabolic, cardiovascular, and neuropsychiatric disorders later in life (Roseboom *et al*., 2001). These long-term effects are encompassed within the Developmental Origins of Health and Disease (DOHaD) framework, which emphasizes the lasting consequences of early-life environmental influences (Barker *et al*., 1993; Gluckman and Hanson, 2004). Within this framework, fetal brain development has emerged as a particularly sensitive target of the effects of placentally mediated programming; such effects include altered risk of central nervous system disorders, including depression, attention-deficit hyperactivity disorder, schizophrenia, and autism spectrum disorders. Human cohort studies have identified associations between placental and cord blood metabolic profiles and later neurodevelopmental outcomes, including increased autism risk in genetically susceptible populations (Parenti *et al*., 2024). In parallel, maternal stress, infection, and malnutrition during pregnancy have been associated with increased neuropsychiatric risk for the offspring later in life (Bale *et al*., 2010; Brown and Derkits, 2010; Babineau *et al*., 2022; Monk and Fernández, 2022; O’Connor *et al*., 2022; de Leeuw *et al*., 2025). These observations position placental metabolism as a critical intermediary linking maternal health and environment to fetal brain development (Mastorci and Agrimi, 2019).

Among the placental metabolic pathways involved in fetal brain programming, placental tryptophan metabolism has gained increased attention because it generates bioactive metabolites, including serotonin (5-hydroxytryptamine; 5-HT), melatonin, and kynurenines, that regulate immune tolerance, oxidative balance, vascular function, and neurodevelopmental signaling (Badawy, 2015). Emerging evidence suggests that disruptions in placental tryptophan metabolism contribute to the developmental origins of mental health disorders and adverse pregnancy outcomes (Goeden *et al*., 2013). Importantly, regulation of this pathway is established early in pregnancy, during periods of rapid placental differentiation and embryonic development, and continues to adapt across gestation. Maternal cardiometabolic disorders, inflammatory conditions, and early pregnancy complications can disrupt this pathway, and such alterations have been associated with pregnancy loss and later pathologies, including preeclampsia, fetal growth restriction (FGR), and preterm birth (Goeden *et al*., 2013). Recent studies further implicate periconceptual and early gestational tryptophan metabolism in shaping embryonic growth trajectories, placental vascular development, and susceptibility to hypertensive disorders of pregnancy (van Zundert *et al*., 2024a,b,c). In this review, we synthesize the current evidence on placental tryptophan metabolism across gestation, with attention given to early regulatory events and subsequent gestational adaptation. We discuss physiological dynamics during healthy pregnancy and the impact of maternal inflammatory, hypoxic, oxidative, and metabolic stressors on the 5-HT/melatonin and kynurenine branches of tryptophan catabolism. Finally, we consider experimental models used to study placental tryptophan metabolism and transport, highlighting methodological considerations critical for gestational stage-specific interpretation.

## Methods

A comprehensive literature search was conducted using the PubMed database to identify studies relevant to placental tryptophan metabolism and its implications for fetal development. The following keywords and their combinations were used: ‘placenta’, ‘tryptophan metabolism’, ‘serotonin’, ‘melatonin’, ‘kynurenine’, ‘fetal programming’, ‘neurodevelopment’, and ‘Developmental Origins of Health and Disease (DOHaD)’. No time or species restrictions were applied to maximize coverage of the topic. Only full-text, peer-reviewed articles published in English were included. Abstracts, conference proceedings, and non-peer-reviewed literature were excluded to ensure data reliability. The search covered literature available up to May 2025. Additional references were identified by manually screening the bibliographies of key articles.

## Tryptophan availability and placental transport

**Figure 1.**
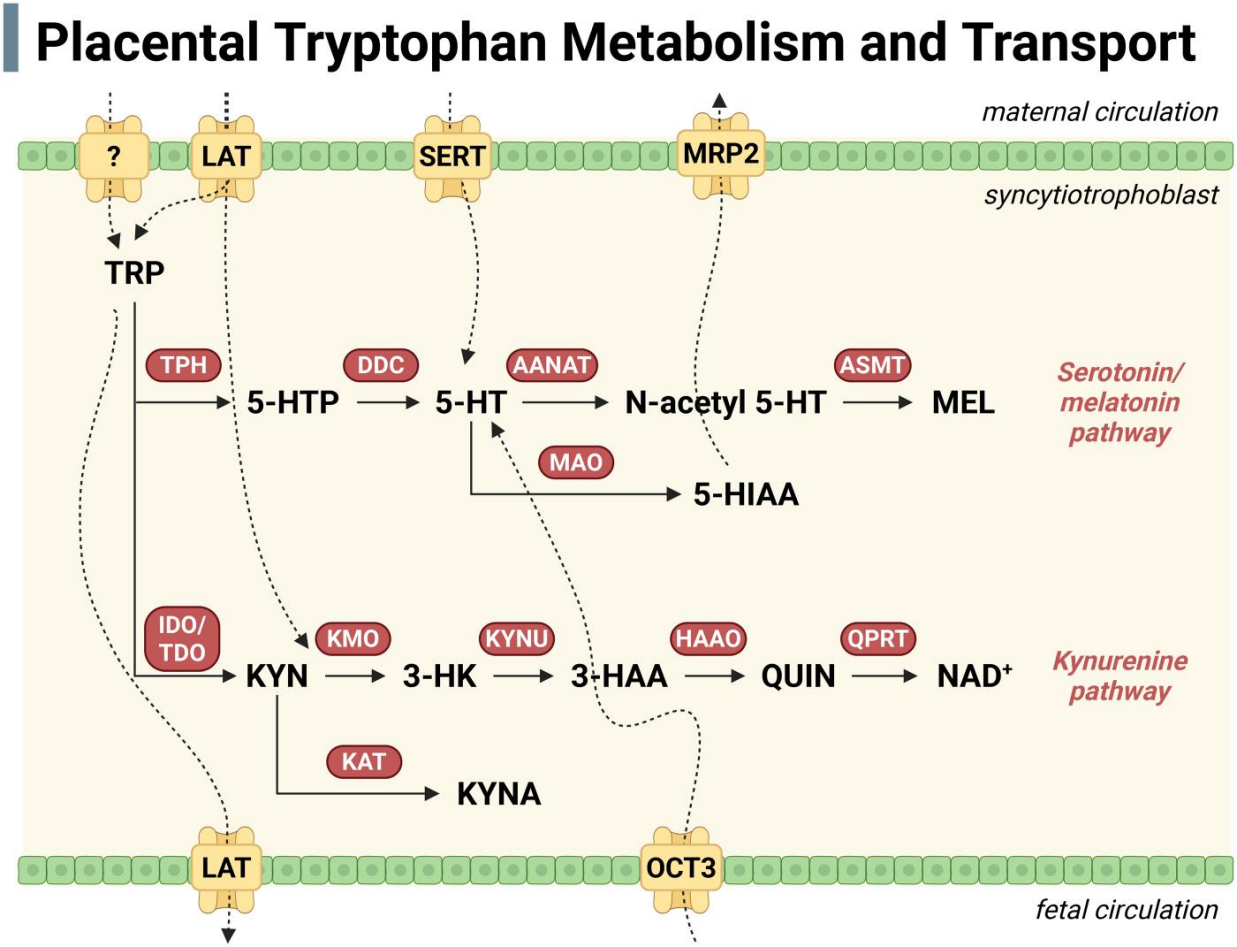
**General schematic of tryptophan metabolism and transport in the placenta.** This diagram illustrates the major metabolic pathways of tryptophan, including its conversion to serotonin, melatonin, and kynurenine, along with key enzymes and transporters involved. The localization of enzymes shown reflects a simplified overview, primarily in syncytiotrophoblasts, and does not account for dynamic spatial changes that occur across gestation. For a detailed characterization of cell-specific localization across gestation, see previous literature (Broekhuizen *et al*., 2021; Perić *et al*., 2022). 3-HAA, 3-hydroxy anthranilic acid; 3-HK, 3-hydroxykynurenine; 5-HIAA, 5-hydroxyindoleacetic acid; 5-HT, serotonin; 5-HTP, 5-hydroxytryptophan; AANAT, aralkylamine *N*-acetyltransferase; ASMT, *N*-acetylserotonin O-methyltransferase; DDC, dihydroxyphenylalanine (DOPA) decarboxylase; HAAO, hydroxyanthranilate 3,4-dioxygenase; IDO, indoleamine 2,3-dioxygenase; KAT, kynurenine aminotransferase; KMO, kynurenine monooxygenase; KYN, kynurenine; KYNA, kynurenic acid; KYNU, kynureninase; LAT, system L amino acid transporter; MAO, monoamine oxidase; MEL, melatonin; MRP2, multidrug resistance-associated protein 2; NAD, nicotinamide adenine dinucleotide; OCT3, organic cation transporter 3; QPRT, quinolinate phosphoribosyl transferase; QUIN, quinolinic acid; SERT, serotonin transporter; TDO, tryptophan 2,3-dioxygenase; TPH, tryptophan hydroxylase; TRP, tryptophan. Created in BioRender https://BioRender.com/x4tu03g.

Tryptophan is an essential amino acid required for multiple physiological processes and is obtained exclusively from dietary sources such as meat, fish, dairy products, eggs, legumes, nuts, and seeds. Only a small fraction (∼5%) of dietary tryptophan is incorporated into protein, with most serving as precursors for bioactive metabolites generated via the 5-HT/melatonin, kynurenine, and indole pathways (Badawy, 2019). During pregnancy, tryptophan demand increases to support fetal and placental growth and the production of metabolites relevant to fetal development and programming (Badawy, 2015, 2017). In the placenta, the 5-HT/melatonin and kynurenine pathways are the most mechanistically established and best characterized (Badawy, 2015). Indole-derived metabolites, including indole-3-lactic acid, have been reported to be elevated in preeclampsia and may influence endothelial and trophoblast function via aryl hydrocarbon receptor signaling (Zhao *et al*., 2022b; Wei *et al*., 2025). However, placental handling of indole metabolites remains poorly defined; therefore, this review focuses on the 5-HT/melatonin and kynurenine pathways (Fig. 1).

Adequate maternal tryptophan availability is critical from early pregnancy onward, as both the placenta and fetus rely entirely on the maternal supply. Availability is largely determined by circulating free tryptophan, which is influenced by nutritional status, hormonal milieu, psychological stress, and pharmacological factors (Badawy, 2010). Human and experimental rodent studies show that although total plasma tryptophan concentrations decline during pregnancy, free tryptophan levels actually increase. This increase is attributed to reduced serum albumin concentrations, which normally bind most circulating tryptophan, together with elevated levels of non-esterified fatty acids that displace tryptophan from albumin (Badawy, 2015). Increased free tryptophan, therefore, enhances the fraction available for placental uptake and metabolism during gestation.

Maternal tryptophan availability alone does not determine fetal exposure. Placental transport of tryptophan represents a critical rate-limiting step that governs placental content, downstream metabolism, and fetal supply (Kudo and Boyd, 2001). Transplacental transport occurs primarily via the system L amino acid transporters (LATs) expressed in the placental syncytiotrophoblast (Ganapathy *et al*., 1986; Kudo and Boyd, 2001). Uptake across the apical membrane is mediated by LAT1 (*SLC7A5*), which forms a functional heterodimer with CD98 (*SLC3A2*), which is required for membrane localization and transport activity (Ganapathy *et al*., 1986; Kudo and Boyd, 2001). Basal membrane transport is less well defined but likely involves LAT2 (*SLC7A8*) and CD98 interactions with components of the y + L system (Kudo and Boyd, 2001). Additional high-affinity tryptophan-specific transporter systems have been suggested but remain to be fully characterized (Kudo and Boyd, 2001; Seymour *et al*., 2006; Bhutia *et al*., 2015). In line with adaptive placental regulation, we reported that placental tryptophan content and *Slc7a5/Slc7a8* expression increase with advancing gestation in rats (Abad *et al*., 2020), supporting increased transport capacity to meet growing fetal and placental demands. Whether similar gestational adaptations occur in the human placenta remains to be established.

Beyond availability and transport, the partitioning of tryptophan between metabolic pathways varies across gestation and in response to maternal signals. Outside pregnancy, increased kynurenine pathway activity is often accompanied by reduced 5-HT synthesis (and vice versa), consistent with competition for a shared substrate (Rizza *et al*., 1983; Oxenkrug, 2013). In pregnancy, and particularly in placental tissue, this reciprocal relationship is less consistent and appears to diverge from early gestation onward (van Zundert *et al*., 2024c). Rather than simple substrate competition, pathway activity is shaped by regulators, including inflammation, infection, and steroid hormones, whose levels and effects vary across gestation. Glucocorticoids (Jovanovic *et al*., 2023), as well as estrogen and progesterone (Sha *et al*., 2021), modulate both the kynurenine and 5-HT pathways, often enhancing kynurenine synthesis while limiting 5-HT production. Maternal stress may selectively affect placental serotonergic signaling without affecting kynurenine metabolism (Melnikova *et al*., 2024), highlighting distinct regulatory sensitivities. Together, the early divergence of placental 5-HT and kynurenine pathway activity suggests that regulation of placental tryptophan metabolism is already established in early pregnancy and continues to shape fetal exposure to downstream metabolites throughout gestation.

## The 5-HT and melatonin pathways

Tryptophan metabolism along the 5-HT pathway generates 5-HT and melatonin, which support key neuroendocrine and placental adaptations in pregnancy (Iwasaki *et al*., 2005; Napso *et al*., 2018). Within the placenta, 5-HT and melatonin have been linked to processes that shape placental development and function and, in parallel, influence fetal organ development and developmental programming (Bonnin *et al*., 2007; Nagai *et al*., 2008; Bonnin and Levitt, 2011; Perić *et al*., 2022). Beyond the placenta, these metabolites are associated with broader maternal adaptations, including glucose homeostasis, steroidogenesis, and lactation (Hudon Thibeault *et al*., 2017; Napso *et al*., 2018), while melatonin additionally contributes to circadian rhythmicity (Bates and Herzog, 2020). Together, these observations place the 5-HT–melatonin branch among the pathways most relevant during early gestation, when placental differentiation and fetal organogenesis are actively underway.

### Placental 5-HT biosynthesis, metabolism, and transport

5-HT biosynthesis is initiated by tryptophan hydroxylase (TPH), the rate-limiting enzyme that converts tryptophan to 5-hydroxytryptophan (5-HTP), which is subsequently decarboxylated to 5-HT by aromatic L-amino acid decarboxylase (dihydroxyphenylalanine (DOPA) decarboxylase) (Fig. 1). Of the two TPH isoforms, TPH2 is largely restricted to the central nervous system (Alenina *et al*., 2009), whereas TPH1 is expressed in several peripheral tissues, including the placenta (Côté *et al*., 2003; Matsuda *et al*., 2004; Bonnin *et al*., 2011). 5-HT is metabolized to 5-hydroxyindoleacetic acid (5-HIAA) by monoamine oxidase (MAO), which has two isoforms: MAO-A and MAO-B. MAO-A preferentially metabolizes 5-HT and is the dominant isoform in the placenta (Sivasubramaniam *et al*., 2002; Shih *et al*., 2011). Placental 5-HT transport is mediated by the high-affinity, low-capacity 5-HT transporter (serotonin transporter [SERT]/*SLC6A4*) and the low-affinity, high-capacity organic cation transporter 3 (OCT3/*SLC22A3*), which are functionally expressed on the maternal and fetal sides, respectively (Balkovetz *et al*., 1989; Sata *et al*., 2005; Karahoda *et al*., 2020b) (Fig. 1).

### Placental supply of 5-HT to the fetal brain in early pregnancy

**Figure 2.**
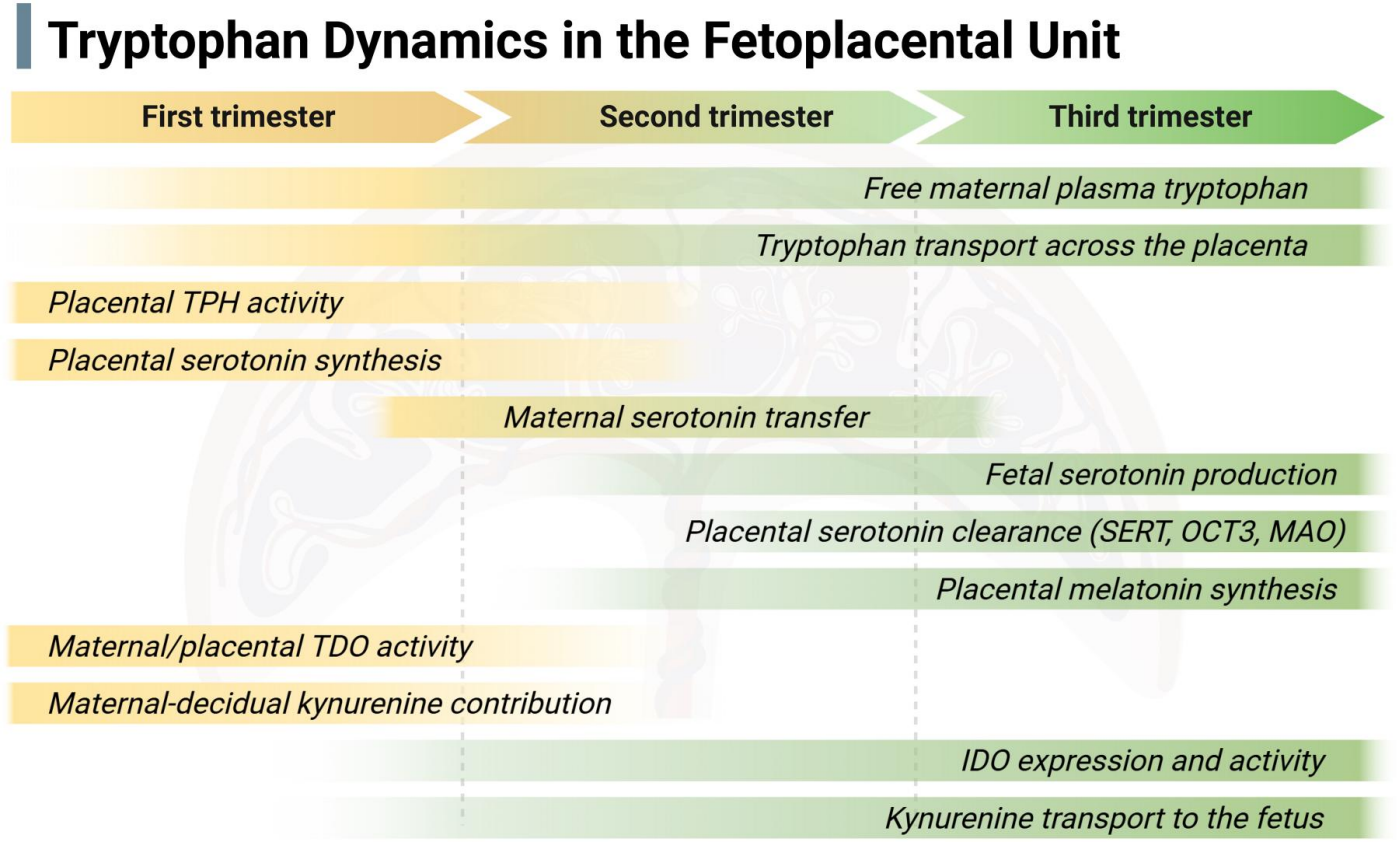
**Proposed dynamic changes in tryptophan pathways during gestation.** Hypothesized shifts in the expression and activity of key enzymes and transporters involved in placental tryptophan metabolism across the three trimesters of gestation. Proposed changes highlight the metabolic transitions that may influence the balance between serotonin, melatonin, and kynurenine pathway metabolites throughout pregnancy. The scheme is based on available evidence from both human and animal studies; however, species-specific differences should be taken into account when interpreting the figure. IDO, indoleamine 2,3-dioxygenase; MAO, monoamine oxidase; OCT3, organic cation transporter 3; SERT, serotonin transporter; TDO, tryptophan 2,3-dioxygenase; TPH, tryptophan hydroxylase. Created in BioRender https://BioRender.com/04yulxk.

A defining feature of serotonergic regulation during pregnancy is its marked developmental timing (Fig. 2). Expression mapping has revealed 5-HT receptor transcripts in the early fetal brain before the establishment of intrinsic serotonergic innervation (Bonnin *et al*., 2006), raising the question of how 5-HT becomes available to the fetus during early gestation. Maternal 5-HT was initially proposed as a potential source, supported by placental expression of SERT on the apical membrane, which facilitates maternal 5-HT uptake into the placental tissue (Prasad *et al*., 1996). Furthermore, maternal alterations in 5-HT synthesis or signaling, including deficiency of TPH1, loss of 5-HT_1A_ receptor signaling, or expression of an autism-associated SERT variant, have been linked to abnormalities of fetal development (Cote *et al*., 2007; Gleason *et al*., 2010; Muller *et al*., 2017). However, fetal forebrain 5-HT concentrations remain preserved in offspring of SERT-deficient dams (Chen *et al*., 2001; Bonnin *et al*., 2011), and *ex vivo* placental perfusion studies in mouse (Bonnin *et al*., 2011), rat (Karahoda *et al*., 2020b), and human (Mathiesen *et al*., 2020) placentas demonstrate minimal transfer of free maternal 5-HT into the fetal circulation. Together, these findings indicate that maternal 5-HT is unlikely to represent the primary fetal source during early pregnancy and point instead to a central role for the placenta itself.

Bonnin *et al*. (2011) demonstrated that the murine placenta during early gestation can synthesize 5-HT *de novo* from maternal tryptophan via placental TPH activity. This finding provided a mechanistic explanation for how 5-HT becomes available to the fetal brain before maturation of the fetal central serotonergic neurons. Subsequent studies confirmed placental 5-HT synthesis in primary trophoblast cells isolated from the human term placenta (Laurent *et al*., 2017), indicating that the enzymatic machinery required for placental 5-HT production is conserved in humans. Functional studies in mice further revealed that placental 5-HT is delivered to the fetal forebrain within a defined developmental window, where it influences cortical neurogenesis, neuronal migration, and early axon targeting (Gaspar *et al*., 2003; Bonnin *et al*., 2011). Collectively, these findings demonstrate that the placenta functions as an active neuroendocrine organ from early pregnancy, with a temporally restricted window of placental 5-HT delivery coinciding with key stages of fetal brain patterning and placental differentiation.

### Transition from placental to fetal 5-HT synthesis across gestation

In mice, placental 5-HT delivery to the fetal forebrain occurs primarily between embryonic days E10 and E15, corresponding to an early gestational window in humans. After this period, 5-HT availability increasingly depends on endogenous fetal sources. This transition is supported by declining forebrain 5-HT levels in Pet-1-deficient mice after E16, indicating that 5-HT detected later in gestation originates predominantly from embryonic serotonergic neurons (Bonnin *et al*., 2011). Consistent with this shift, expression of serotonergic biosynthetic machinery emerges in fetal tissues as gestation advances. *Tph2* expression is detectable in enteric neurons by E12.5 (Cote *et al*., 2007), while *Tph1* expression appears in enterochromaffin cells by E15.5, with endogenous 5-HT detected in these cells by E16 (Branchek and Gershon, 1989). 5-HT derived from these sources enters the fetal circulation and accounts for the majority of circulating fetal 5-HT at later gestational stages (Chen *et al*., 2001). In parallel, active transport of maternal tryptophan across the placenta persists throughout gestation, supporting fetal 5-HT synthesis from this maternal supply of substrate (Sano *et al*., 2016; Karahoda *et al*., 2020b).

We further examined fetal serotonergic synthesis capacity in rats and found that *Tph1* transcript levels in the fetal intestine, brain, lungs, and liver during late gestation exceeded those observed in the term placenta, although functional TPH activity was confined to the fetal brain and intestine (Abad *et al*., 2020). These findings align with earlier observations of fetal brain 5-HT synthesis near term (Arevalo *et al*., 1991; Sano *et al*., 2016) and support the concept that placental 5-HT output declines as fetal serotonergic systems mature. This is further supported by reduced placental TPH expression, lower placental 5-HTP levels, and a declining 5-HTP/tryptophan ratio toward term (Abad *et al*., 2020).

### 5-HT clearance at the fetal interface in late gestation

Given the dynamic regulation of fetal 5-HT across gestation, mechanisms that limit serotonergic exposure become increasingly important, particularly in late pregnancy. In adults, platelets serve as a major 5-HT reservoir; however, the contribution of platelet storage in the fetal circulation remains unclear. Early studies suggested that fetal platelets are less abundant and functionally immature compared with adult platelets (Ts’ao *et al*., 1976), a conclusion supported by more recent work showing reduced platelet numbers and impaired platelet function in the developing mouse fetus (Margraf *et al*., 2017). In humans, platelet 5-HT levels are also low at birth (Anderson *et al*., 2004), indicating that platelet-mediated 5-HT storage is unlikely to represent a major regulatory mechanism during fetal life.

To identify alternative regulatory pathways, we examined the activity of MAO in fetal rat and placental tissues. Significant MAO activity was detected in both the placenta and fetal rat brain during prenatal development (Abad *et al*., 2020), aligning with earlier findings emphasizing the pivotal role of placental MAO in 5-HT metabolism (Lewinsohn *et al*., 1980; Kono *et al*., 1991). This supports the view that placental–fetal enzymatic clearance, rather than platelet sequestration, plays a central role in regulating 5-HT levels in late gestation.

This concept aligns with the longstanding ‘monoamine clearance’ theory, first proposed in the 1960s (Koren *et al*., 1965, 1966) and later revisited in the 1990s (Ganapathy *et al*., 1993). It posits that placental trophoblasts protect the uteroplacental circulation from excessive vasoconstriction by actively taking up monoamines such as norepinephrine, dopamine, and 5-HT for subsequent metabolism by MAO. A major limitation of early monoamine clearance studies is that they focused primarily on maternal-facing transfer and did not directly assess 5-HT handling at the fetal interface. In mice, fetal and placental 5-HT levels rise in parallel from mid-gestation while maternal levels remain unchanged (Robson and Senior, 1964), supporting the relevance of placental clearance from the fetal side in late pregnancy.

Building on this framework, work from our laboratories demonstrated that 5-HT is actively extracted from the fetal circulation into trophoblast cells via the low-affinity, high-capacity organic cation transporter OCT3 (Karahoda *et al*., 2020b; Staud *et al*., 2023). This transport is concentration-dependent and can be inhibited by endogenous regulators such as cortisol and by exogenous compounds including antidepressant drugs. Consistent with a late-gestation clearance function, OCT3 and SERT expression increase toward term in both rat and human placentas (Lee *et al*., 2013; Abad *et al*., 2020; Karahoda *et al*., 2020a), alongside upregulation of placental MAO-A, thereby enhancing serotonergic degradation capacity (Chen *et al*., 1976; Kono *et al*., 1994; Wu *et al*., 2016; Abad *et al*., 2020; Karahoda *et al*., 2020a).

Together, these findings support a model in which the term placenta functions as an active 5-HT clearance system (Fig. 2). In contrast to early gestation, 5-HT is extracted from the fetal circulation via OCT3, metabolized to 5-HIAA by MAO-A within the trophoblast layer, and subsequently exported to the maternal circulation via multidrug resistance-associated protein 2 (Staud *et al*., 2023). This coordinated transport–metabolism axis is supported by the co-localization of OCT3, SERT, and MAO-A in the syncytiotrophoblast (Verhaagh *et al*., 2001; Ahmadimoghaddam *et al*., 2012; Abad *et al*., 2020) and by preferential maternal secretion of 5-HIAA in human placenta perfusion studies (Mathiesen *et al*., 2020; Staud *et al*., 2023). This system limits excessive fetal serotonergic exposure and supports vascular and neurodevelopmental homeostasis in late pregnancy.

### Placental melatonin synthesis and signaling during pregnancy

Melatonin is produced from 5-HT by the sequential actions of aralkylamine *N*-acetyltransferase (AANAT) (Weissbach *et al*., 1960) and *N*-acetylserotonin O-methyltransferase (ASMT) as shown in Fig. 1 (Axelrod and Weissbach, 1960). While its synthesis in the pineal gland is well-documented, the placenta also contains the enzymatic machinery for local melatonin production (Lanoix *et al*., 2008). Notably, AANAT and ASMT expression is detectable in the human placenta as early as the first trimester and increases across gestation, peaking in the third trimester (Soliman *et al*., 2015) (Fig. 2). This pattern parallels the progressive rise in maternal circulating melatonin during pregnancy, which peaks in late gestation and declines rapidly after delivery (Ejaz *et al*., 2021; Gomes *et al*., 2021).

Melatonin receptors (MT1 and MT2) are expressed in the placenta throughout gestation (Soliman *et al*., 2015). *In vitro*, melatonin enhances syncytium formation and β-hCG secretion, implying that it has a functional role in promoting trophoblast differentiation and endocrine function that supports embryonic and fetal growth (Iwasaki *et al*., 2005; Soliman *et al*., 2015). Together, these findings suggest that melatonin may contribute to placental maturation and adaptation, with potential relevance from early pregnancy onward; however, the specific placental processes governed by locally produced melatonin remain incompletely defined.

Unlike the maternal and placental compartments, the fetus does not produce melatonin during gestation; the fetal pineal gland remains immature and inactive until after birth, so endogenous melatonin synthesis begins only postnatally (Reiter *et al*., 2014; Voiculescu *et al*., 2020). The fetus thus depends entirely on maternal melatonin, which freely crosses the placenta (Okatani *et al*., 1998) and plays a critical role in regulating fetal circadian rhythms and supporting neurodevelopment (Reiter *et al*., 2014; Gomes *et al*., 2021). However, the role of placentally synthesized melatonin in fetal physiology remains unclear and warrants further study.

## The kynurenine pathway

Tryptophan metabolism through the kynurenine pathway generates kynurenine and a series of downstream metabolites, including 3-hydroxykynurenine (3-HK), kynurenic acid (KYNA), and quinolinic acid (QUIN), which exert diverse biological effects during pregnancy. These metabolites play key roles in immune regulation, vascular function, redox balance, and neurodevelopment (Sedlmayr *et al*., 2014; Badawy, 2015). Several kynurenine pathway products directly modulate *N*-methyl-D-aspartate (NMDA) receptor signaling, most notably the neuroprotective antagonist KYNA and the neurotoxic agonist QUIN, thereby influencing neuronal excitability and developmental trajectories (Schwarcz *et al*., 2012; Schwarcz, 2016).

### Placental kynurenine biosynthesis, metabolism, and transport

The greater part of tryptophan is metabolized to kynurenine by one of two rate-limiting enzymes, tryptophan 2,3-dioxygenase (TDO) or indoleamine 2,3-dioxygenase (IDO), depending on the tissue and physiological conditions (Sedlmayr *et al*., 2014). TDO exhibits high affinity and specificity for tryptophan, and, apart from the placenta, is predominantly expressed in the liver, where it serves as the principal regulator of systemic tryptophan levels. However, TDO is also active in extrahepatic tissues, including the kidney and immune cells (Badawy, 2019). IDO exists as two isoforms, IDO1 and IDO2, which differ in catalytic efficiency, expression patterns, and physiological relevance (Ball *et al*., 2007). The human placenta expresses both isoforms, with IDO1 being dominant (Sedlmayr *et al*., 2002; Blaschitz *et al*., 2011; Murthi *et al*., 2017b; Wakx *et al*., 2018; Karahoda *et al*., 2020a).

Kynurenine serves as a substrate for multiple downstream enzymes (Fig. 1). Kynurenine aminotransferases generate KYNA (Schwarcz, 2016). In contrast, kynurenine monooxygenase (KMO) converts kynurenine into 3-HK, which is subsequently metabolized to 3-hydroxyanthranilic acid (3-HAA) and ultimately QUIN (Schwarcz *et al*., 2012). The balance between KYNA and QUIN is critical for NMDA receptor signaling (Schwarcz, 2016), and shifts in this ratio, particularly under pathological conditions, may have significant implications for fetal brain development and long-term neurodevelopmental risk. Given the biological activity of these metabolites, we and others have shown that the complete enzymatic machinery of the kynurenine pathway is expressed in both human and rat placentas, highlighting the placenta’s role in producing these metabolites during gestation (Manuelpillai *et al*., 2003; Abad *et al*., 2020; Karahoda *et al*., 2020a). This is particularly important given that placenta-derived kynurenine metabolites have been implicated in the pathogenesis of perinatal brain injury (Goeden *et al*., 2017).

The biological activity of kynurenine pathway metabolites necessitates tight regulation of their distribution across tissues and cellular compartments. Kynurenine and its derivatives are transported by multiple membrane transporters, and intercellular exchange of these metabolites (analogous to mechanisms described in the brain) likely contributes to local pathway regulation (Guillemin *et al*., 2001). LAT1/*SLC7A5* is the key transporter for kynurenine uptake (Fukui *et al*., 1991), while KYNA can be transported across barriers by organic anion transporters 1 and 3 (OAT1/*SLC22A6* and OAT3/*SLC22A8*) (Uwai *et al*., 2012). 3-HK shares transporters with tryptophan and kynurenine, such as large neutral amino acid transporters (Okuda *et al*., 1998), whereas QUIN is transported by the Na^+^-dependent excitatory amino acid transporter 3 (EAAT3/*SLC1A1*) (Braidy *et al*., 2021).

### Maternal–decidual kynurenine pathway activity in early pregnancy

Recent evidence indicates that kynurenine pathway activity is present very early in pregnancy, preceding the establishment of full placental perfusion and metabolic capacity. Kynurenine pathway metabolites have been detected in the maternal circulation as early as 7–10 weeks of gestation (van Zundert *et al*., 2022, 2024c). At this developmental stage, the embryo is supported primarily by histotrophic nutrition from the uterine glands (Burton *et al*., 2001), suggesting that early kynurenine metabolites originate largely from maternal endometrial or uterine vascular sources. These metabolites may therefore be secreted into the coelomic fluid and taken up by the developing trophectoderm, indicating that kynurenine pathway activity is already integrated into the maternal–embryonic interface before full placental maturation.

Mechanistically, this early activity is consistent with gestation-specific regulation of IDO expression and hormonal signaling at the maternal–decidual interface. Isoform-specific studies show that IDO1 is expressed in the decidual glandular epithelium in early gestation, while its expression later shifts toward decidual macrophages and placental endothelial cells at term (Kudo *et al*., 2020; Murthy *et al*., 2021; Broekhuizen *et al*., 2021). In contrast, IDO2 is expressed in syncytiotrophoblasts throughout gestation, with additional expression in extravillous trophoblasts (Broekhuizen *et al*., 2021), suggesting distinct and potentially complementary roles for the two isoforms across pregnancy. Importantly, vascular endothelial cells are known to express inducible IDO under inflammatory and hormonal stimuli and to produce kynurenine as a regulator of vascular tone (Wang *et al*., 2010). This is particularly relevant in early pregnancy, when progesterone levels are high due to corpus luteum activity (Sha *et al*., 2021). Progesterone has been shown to modulate kynurenine pathway enzymes, including IDO activity (Jovanovic *et al*., 2023), and may therefore promote kynurenine production within the uterine vasculature. Through its vasodilatory and immunomodulatory effects, endothelial IDO-mediated kynurenine production may contribute to early uterine vascular adaptation, supporting increased perfusion and immune tolerance as the placenta develops. Collectively, these findings support the concept of a model in which early kynurenine pathway activity is hormonally and vascularly regulated at the maternal–decidual interface, preceding and then complementing placental kynurenine metabolism later in gestation.

### Gestational shifts in placental kynurenine metabolism and immune function

As pregnancy advances and the placenta establishes its full functional capacity, the site of primary kynurenine production appears to shift progressively toward the maternal–fetal interface. This transition has historically been interpreted through the concept of IDO1-mediated immune tolerance, which emerged from experimental work by Munn *et al*. (1998). In that study, pharmacological inhibition of IDO activity using 1-methyltryptophan in pregnant mice led to rejection of allogeneic conceptuses, accompanied by increased maternal T-cell activation. These findings were interpreted as evidence that placental IDO1 suppresses maternal immune responses by depleting local tryptophan, thereby limiting T-cell proliferation and effector function at the maternal–fetal interface. Together with the demonstration of IDO expression in the human syncytiotrophoblast, this work established a widely accepted model in which placental IDO1-mediated tryptophan catabolism is central to maternal–fetal immune tolerance.

However, subsequent studies have revealed important limitations in this interpretation. The IDO inhibitor used in the original experiments, 1-methyltryptophan, was later shown to inhibit placental tryptophan transport independently of IDO enzymatic activity (Kudo and Boyd, 2001; O’Connor and Green, 2013). This raises the possibility that immune effects attributed to enzymatic tryptophan degradation may, at least in part, reflect altered substrate availability due to transporter inhibition. Second, the C57BL/6J mouse strain used in early experiments has unusually high basal TDO activity and therefore relatively low free and total serum tryptophan levels (Badawy, 2015). This metabolic background may amplify the apparent effects of further tryptophan restriction and complicate attribution of immune suppression specifically to placental IDO1. Moreover, although total plasma tryptophan concentrations decline, free tryptophan levels increase, challenging the ‘tryptophan depletion’ model *in vivo* (Badawy, 2015).

Genetic studies further suggest that IDO is not strictly required for pregnancy maintenance. Allogeneic pregnancies in IDO-deficient mice progress normally and retain viable embryos (Baban *et al*., 2004), and loss of IDO function in pregnant mice does not compromise fetal survival (Santillan *et al*., 2015). These findings suggest that compensatory pathways can support immune tolerance in the absence of IDO activity. These may include alternative enzymatic routes of tryptophan metabolism, such as TDO, or parallel immunoregulatory mechanisms independent of tryptophan depletion.

Consistent with this view, we reported preferential expression of downstream kynurenine pathway enzymes in first-trimester human placentas despite minimal placental IDO expression at this stage (Karahoda *et al*., 2020a). Given that placental TDO expression remains relatively stable across gestation (Abad *et al*., 2020; Karahoda *et al*., 2020a), these findings support the interpretation that early kynurenine pathway flux depends predominantly on TDO-driven kynurenine production and/or maternal-derived kynurenines. As gestation progresses, expansion of placental vascular and immune compartments is accompanied by increased IDO1 expression, suggesting a progressively greater contribution of IDO1 to placental tryptophan metabolism toward term (Sedlmayr *et al*., 2002; Blaschitz *et al*., 2011; Murthi *et al*., 2017b; Wakx *et al*., 2018; Karahoda *et al*., 2020a). Together with previous literature, these data support Badawy’s (2015) hypothesis that placental tryptophan degradation in early to mid-pregnancy is primarily catalyzed by TDO, with IDO playing a more limited or context-dependent role from mid-gestation onward. The contribution of downstream kynurenine metabolites to allogeneic fetal tolerance remains incompletely defined and warrants further investigation, particularly given evidence that several metabolites within this pathway can exert immunosuppressive effects on T-cell function (Badawy, 2019).

Beyond enzymatic expression and immunomodulatory models, functional measurements of kynurenine availability provide additional insight into how placental control of this pathway evolves across gestation. We recently reported that placental kynurenine levels in rats decline significantly toward term (Abad *et al*., 2020), a change that may reflect increased downstream metabolism within the placenta and/or altered transport to the fetal circulation as placental capacity matures. Consistent with a placental shift toward greater metabolic control later in pregnancy, IDO activity at term was highest in the placenta, whereas fetal organs showed limited capacity for kynurenine production. Although the fetal liver exhibited relatively high *Ido2* transcript levels, corresponding IDO enzymatic activity was low (Abad *et al*., 2020). Together with earlier reports demonstrating undetectable TDO activity in fetal and neonatal rat livers (Roper and Franz, 1977), these findings indicate that fetal tissues may not be fully equipped to generate kynurenine during gestation, at least in rodents. Collectively, these findings support that in the rodent model, the placental kynurenine metabolism is dynamically reprogrammed across gestation (Fig. 2), transitioning from maternal IDO- and TDO-dominated sources early in pregnancy to placental IDO-driven regulation later, with limited compensatory capacity in fetal tissues.

## Factors affecting tryptophan homeostasis during pregnancy

Maternal and environmental factors shape tryptophan availability and metabolism from very early gestation, with potential long-term consequences for placental function and fetal development. Maternal characteristics such as ethnicity, body mass index, and medication use have been shown to influence circulating tryptophan and its metabolites already in the first trimester (van Zundert *et al*., 2024a). While the impact of these factors on the 5-HT pathway has been studied in some detail (van Zundert *et al*., 2024c), their effects on kynurenine pathway activity, including kynurenine, KYNA, and QUIN, remain comparatively underexplored, particularly in early gestation.

Psychosocial stress represents a key early modulator of tryptophan homeostasis. Activation of the maternal hypothalamic–pituitary–adrenal axis increases cortisol levels, which in turn can directly regulate placental transporters and enzymes involved in both the 5-HT and kynurenine pathways (Goeden *et al*., 2013; Mathiesen *et al*., 2020). Consistent with this, maternal depression during pregnancy has been associated with altered circulating levels of tryptophan and its metabolites (Nazzari *et al*., 2020; Liu *et al*., 2022; Sha *et al*., 2022). Nutritional factors also intersect with these pathways early in development. In particular, low vitamin D levels during pregnancy, especially when combined with genetic susceptibility, have been linked to impaired 5-HT synthesis and signaling (Patrick and Ames, 2014, 2015; Murthi *et al*., 2017a). Given the central role of 5-HT in early neurodevelopment, these findings raise the possibility that vitamin D status represents a modifiable determinant of early-life programming.

Environmental exposures further influence placental tryptophan metabolism. Xenobiotics such as pharmaceutical agents, illicit substances, environmental pollutants, and dietary compounds can alter the expression and activity of placental transporters and metabolic enzymes (Goeden *et al*., 2016; Rock *et al*., 2020; Horackova *et al*., 2021; Tan *et al*., 2022; Vachalova *et al*., 2022; Lu *et al*., 2023). Such exposures may limit substrate availability, induce oxidative stress, or disrupt regulatory signaling pathways, thereby reshaping tryptophan catabolism. In parallel, chronic hypoxia, inflammation, and oxidative stress (all hallmarks of pregnancy complications such as preeclampsia, FGR, and preterm birth) are known to interfere with both 5-HT and kynurenine pathway activity, potentially amplifying early metabolic disturbances (see below).

Genetic and epigenetic mechanisms provide an additional layer of regulation linking the maternal environment to placental tryptophan metabolism. Polymorphisms in genes encoding key enzymes and transporters of the 5-HT and kynurenine pathways have been associated with altered metabolite profiles and neuropsychiatric disorders in the general population, including depression, autism spectrum disorders, schizophrenia, and bipolar disorder (Claes *et al*., 2011; Boros *et al*., 2018; Pisanu *et al*., 2022; Xue *et al*., 2023). In the placenta, variants in genes such as *MAO-A* and *SLC6A4* have been linked to differences in expression or activity (Zhang *et al*., 2010), suggesting that inherited variation may modulate placental 5-HT metabolism.

Epigenetic regulation appears to be particularly important during early pregnancy, when placental gene expression patterns are being established. DNA methylation of *IDO1* provides a well-characterized example: Spinelli *et al*. (2019) demonstrated that *Ido1* and *Ido2* are maternally expressed and subject to imprinting in the mouse placenta, with the paternal allele being hypermethylated. Aberrant methylation of the *Ido1* promoter was associated with pregnancy loss in a spontaneous abortion mouse model, further supporting the notion that dysregulation of this pathway contributes to poor outcomes. In humans, *IDO1* shows partial methylation in placental tissue, with increased methylation observed in placentas from early pregnancy loss compared with control pregnancies, indicating conserved epigenetic sensitivity across species (Spinelli *et al*., 2019). Similar regulatory principles apply to the 5-HT pathway; for example, methylation of the *SLC6A4* promoter is responsive to the maternal metabolic milieu, with gestational diabetes mellitus (GDM) associated with reduced methylation and increased placental SERT/*SLC6A4* expression (Blazevic *et al*., 2017). This indicates that maternal glucose levels may epigenetically reprogram 5-HT transport capacity, potentially affecting fetal 5-HT exposure during time-sensitive developmental windows.

Beyond individual loci, emerging epigenomic studies suggest that maternal inflammation, obesity, and metabolic stress can broadly reprogram genes within the tryptophan–kynurenine pathway. Altered methylation of *IDO1*, *TDO*, and downstream enzymes has been proposed as a mechanism linking adverse maternal environments to shifts in tryptophan catabolism, favoring kynurenine production over 5-HT synthesis (Gharipour *et al*., 2025). Collectively, these findings highlight genetic and epigenetic regulation as critical mechanisms through which early-life exposures shape placental tryptophan metabolism and, ultimately, long-term offspring health.

### Chronic inflammation

Maternal inflammation during pregnancy is increasingly recognized as a major risk factor for adverse neurodevelopmental outcomes in offspring, including psychiatric and cognitive disorders (Rosenberg, 2018). These effects are mediated by pro-inflammatory cytokines that either cross the placenta or activate placental signaling pathways, thereby altering placental function and fetal developmental trajectories (Woods *et al*., 2023). Among placental metabolic systems, both the 5-HT/melatonin and kynurenine pathways are particularly sensitive to inflammatory cues (Manuelpillai *et al*., 2005; Goeden *et al*., 2013).

Pro-inflammatory cytokines such as interferon-γ (IFN-γ), interleukin-6 (IL-6), and tumour necrosis factor-α strongly induce IDO in placental cells (Murthy *et al*., 2021). We recently confirmed this in human placental explants exposed to Poly I:C or lipopolysaccharide (LPS), where inflammatory stimulation markedly increased IDO expression, kynurenine production, and the kynurenine/tryptophan ratio (Abad *et al*., 2024). In parallel, inflammatory conditions upregulated KMO, increased levels of the neurotoxic metabolite QUIN, and reduced concentrations of the neuroprotective metabolite KYNA, resulting in a pronounced shift of the KYNA/QUIN balance toward a neurotoxic profile (Ligam *et al*., 2005; Abad *et al*., 2024).

Inflammation simultaneously disrupts the placental 5-HT pathway. In our explant model, inflammatory stimulation reduced 5-HT synthesis through downregulation of TPH while enhancing 5-HT degradation via upregulation of MAO-A (Abad *et al*., 2024). These changes are of particular concern in early pregnancy, when the fetal brain relies heavily on placental 5-HT supply prior to maturation of fetal serotonergic neurons (Goeden *et al*., 2013, 2016; Nazzari *et al*., 2020; Sha *et al*., 2022). Accordingly, the timing of inflammatory exposure is likely critical: early insults may impair placental 5-HT supply, whereas later inflammation may predominantly affect fetal tryptophan availability and autonomous 5-HT synthesis (Karahoda *et al*., 2020a).

Animal model studies provide strong support for these mechanisms. Maternal immune activation in rodents/rabbits alters placental 5-HT and kynurenine pathway activity (Goeden *et al*., 2016; Williams *et al*., 2017) and reduces maternal melatonin levels, with downstream effects on fetal neurodevelopment (Sérgio Galina Spilla *et al*., 2024). In our most recent work, intra-amniotic LPS exposure in pregnant rats induced robust inflammation accompanied by decreased 5-HT levels in both placenta and fetal brain. Increased 5-HT degradation, upregulation of the kynurenine pathway, and elevated KMO expression also indicated a shift toward a neurotoxic metabolic profile (Abad *et al*., 2025).

Importantly, these experimental observations are corroborated by human data. In a clinical cohort of pregnancies complicated by preterm birth and inflammation, we found that intra-amniotic IL-6 levels correlated strongly with altered expression of placental tryptophan metabolism genes (Karahoda *et al*., 2021). Taken together, these findings identify inflammation-induced reprogramming of placental tryptophan metabolism, particularly during early gestation, as a plausible mechanistic link between maternal immune activation and long-term neurodevelopmental vulnerability in the offspring.

### Chronic hypoxia and oxidative stress

Chronic hypoxia and oxidative stress are central features of pregnancy complications such as preeclampsia and FGR. The placental tryptophan pathway contributes to antioxidant defense by generating metabolites with redox-modulating properties; these include melatonin, KYNA, and xanthurenic acid, which scavenge reactive oxygen species and enhance antioxidant enzyme activity (Jauniaux *et al*., 2006; Xu *et al*., 2018). In addition, the upstream enzymes of this pathway, including IDO and TDO, have been shown to exert intrinsic antioxidant effects (Xu *et al*., 2018).

IDO is a heme-containing, oxygen-dependent enzyme that catalyzes the conversion of tryptophan to *N*-formylkynurenine (Werner and Werner-Felmayer, 2007), rendering its activity particularly sensitive to oxygen availability. In preeclamptic placentas, IDO1 expression and activity are markedly reduced and linked to diminished antioxidant capacity (Santoso *et al*., 2002). Consistent with this, placental IDO1 activity is inversely correlated with levels of 8-hydroxy-2′-deoxyguanosine, indicating that loss of IDO1 function increases oxidative DNA damage. The resulting oxidative lesions are particularly evident in syncytiotrophoblast and placental endothelial cells, sites of high IDO expression in healthy placentas (Nishizawa *et al*., 2011). Beyond redox regulation, IDO1 plays an important role in placental vascular homeostasis. Endothelial IDO1 activity promotes vasodilation through kynurenine-mediated signaling (Wang *et al*., 2010), whereas reduced IDO1 expression in preeclampsia is associated with placental vasoconstriction and impaired perfusion (Zardoya-Laguardia *et al*., 2018).

In pathological hypoxic states such as FGR, reduced oxygen availability may further suppress IDO1 activity, amplifying oxidative stress and limiting tryptophan flux through antioxidant pathways (Murthi *et al*., 2017b). This effect is compounded by inhibition of oxygen-sensitive enzymes downstream of kynurenine, including KMO and 3-hydroxyanthranilate 3,4-dioxygenase (Dang *et al*., 1998, 2000). Suppression of these enzymes is likely to alter the balance of kynurenine metabolites, favoring accumulation of intermediates such as kynurenine and 3-HAA. Consistent with this mechanism, our *ex vivo* studies using placental explants demonstrated that changes in oxygen tension significantly modify the expression of kynurenine pathway enzymes and the release profile of kynurenine metabolites (Murthi *et al*., 2017b). Together, these findings link placental oxygen availability to oxidative stress and tryptophan metabolism. Disruption of this balance during early placental development, when physiological hypoxia gives way to rising perfusion, may promote maladaptation and increase the risk of later complications.

## Pathological disruptions in the placental tryptophan pathway

**Figure 3.**
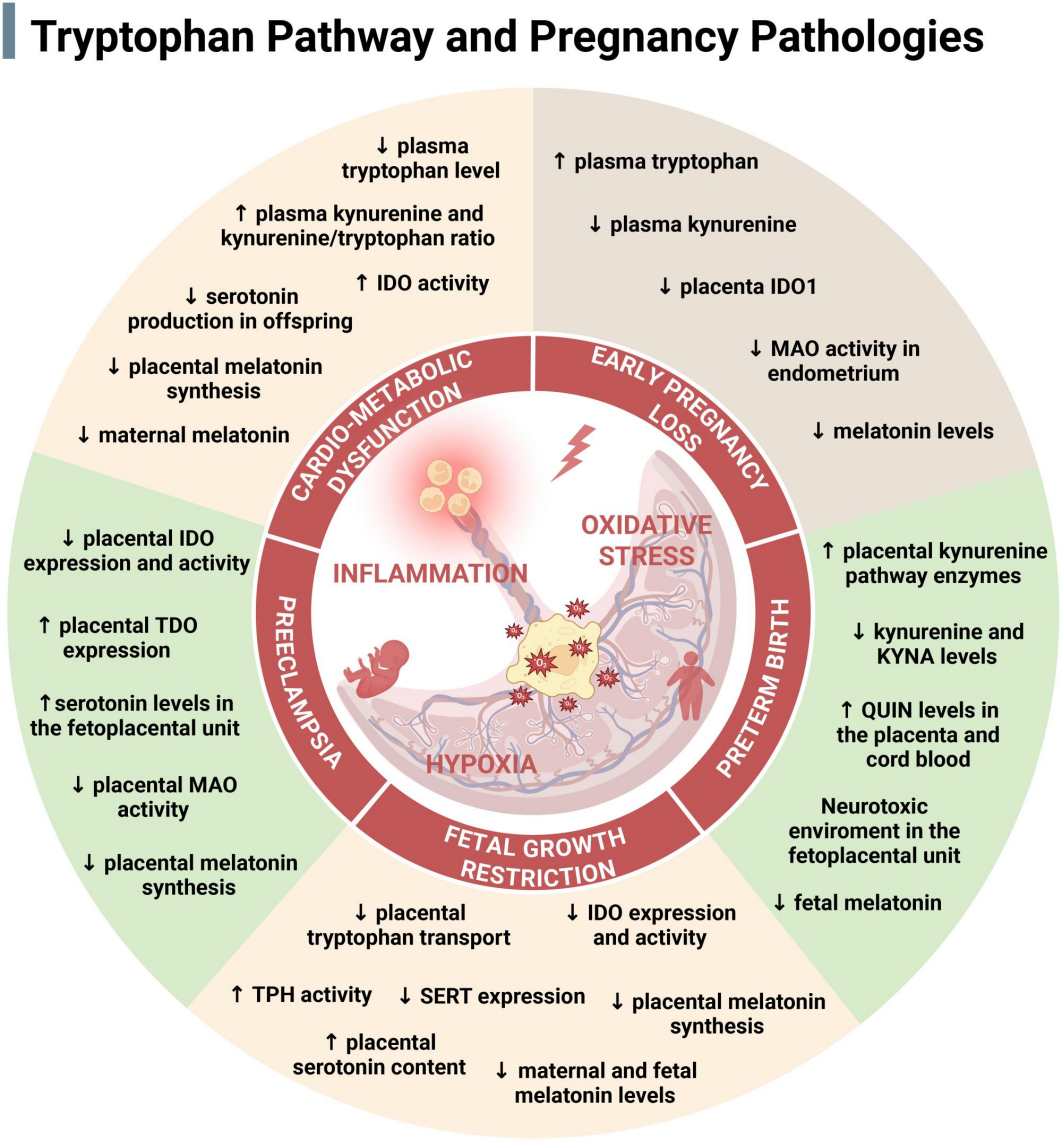
**Alterations in tryptophan metabolism in pregnancy pathologies.** Cardiometabolic dysfunction (including obesity, diabetes, gestational diabetes mellitus, and hypertension), early pregnancy loss (including miscarriage and recurrent miscarriage), preterm birth, fetal growth restriction, and preeclampsia are associated with distinct yet overlapping disruptions in placental tryptophan metabolism. These include alterations in the serotonin, melatonin, and kynurenine pathways, as well as changes in transporter and enzyme expression. Central pathogenic mechanisms, such as inflammation, oxidative stress, and hypoxia, further shape these metabolic responses. The figure summarizes key changes in maternal, placental, and fetal compartments for each pathology, highlighting pathways potentially contributing to impaired placental function, neurodevelopmental programming, and adverse outcomes. IDO, indoleamine 2,3-dioxygenase; KYNA, kynurenic acid; MAO, monoamine oxidase; QUIN, quinolinic acid; SERT, serotonin transporter; TDO, tryptophan 2,3-dioxygenase; TPH, tryptophan hydroxylase. Created in BioRender https://BioRender.com/pelguek.

Placental tryptophan metabolism is highly sensitive to pregnancy pathology, with disruptions emerging early in gestation and often persisting as placental function matures. Maternal cardiometabolic disorders can alter pathway regulation at the maternal–fetal interface, contributing to placental dysfunction and adverse fetal programming. Complications spanning early pregnancy loss (miscarriage and recurrent miscarriage) through to later disorders (preeclampsia, FGR, and preterm birth) further shift the balance between the 5-HT/melatonin and kynurenine branches. Defining these disease-linked signatures across gestation (Fig. 3) may clarify mechanisms and highlight targets to improve pregnancy outcomes.

### Maternal cardiometabolic dysfunction

Maternal cardiometabolic conditions, including obesity, pregestational diabetes, GDM, and hypertension, are increasingly prevalent and represent major risk factors for placental dysfunction and adverse fetal programming. Evidence from rodent models indicates that maternal obesity and high-fat diet exposure impair embryonic serotonergic development, reflected by reduced embryonic 5-HT levels and decreased serotonergic axon density in the developing brain (Jarskog *et al*., 1997; Ishikawa *et al*., 2007; Sullivan *et al*., 2010; Peleg-Raibstein *et al*., 2012; Shook *et al*., 2020). These effects are thought to arise, at least in part, from increased diversion of tryptophan toward the kynurenine pathway, potentially driven by obesity-associated low-grade inflammation that limits 5-HT availability during critical early developmental windows (Sullivan *et al*., 2014; Cussotto *et al*., 2020).

Consistent with this hypothesis, obese individuals, including pregnant women, exhibit reduced circulating tryptophan levels and elevated kynurenine/tryptophan ratios compared with lean controls (Mangge *et al*., 2014; Groer *et al*., 2018; Cussotto *et al*., 2020). Such metabolic shifts may emerge early in pregnancy and have lasting consequences for fetal neurodevelopment. Notably, maternal melatonin supplementation in obese pregnancies has shown protective effects in experimental models, preserving offspring β-cell function, reducing inflammation and oxidative stress, and supporting metabolic homeostasis (Ajackson *et al*., 2023; Lapa Neto *et al*., 2023; Nagagata *et al*., 2023), highlighting the potential of indoleamine modulation during gestation.

Although data on placental tryptophan metabolism during pregnancy in women with pre-existing Type 1 or Type 2 diabetes mellitus remain limited, studies in non-pregnant populations consistently show altered activity of both the 5-HT and kynurenine pathways in these conditions (Oxenkrug, 2013; Unluturk and Erbas, 2015; Gao *et al*., 2023). These metabolic disturbances likely extend to the gestational setting, as diabetes mellitus itself and GDM share key inflammatory and metabolic features (Pervjakova *et al*., 2022; Elliott *et al*., 2024). Accordingly, current insights into gestational tryptophan metabolism derive largely from maternal serum studies, which identify the tryptophan pathway as one of the most consistently altered metabolic networks in GDM pregnancies (Aleidi *et al*., 2024).

Importantly, alterations in tryptophan metabolism are detectable early in gestation. As early as the first trimester, women who later develop GDM exhibit elevated maternal kynurenine levels (McMichael *et al*., 2021), suggesting early pathway dysregulation before clinical diagnosis. Across gestation, pregnancies complicated by GDM show declining maternal serum tryptophan and KYNA levels alongside rising kynurenine, 3-HK, and 3-HAA concentrations, resulting in an increased kynurenine/tryptophan ratio (Özdemir *et al*., 2023). Disruption of the 5-HT–melatonin axis is also evident, with second-trimester samples showing increased 5-HT and reduced melatonin concentrations (Leitner *et al*., 2017; Zheng *et al*., 2017). In parallel, urinary metabolomic studies report elevated tryptophan excretion in GDM pregnancies, consistent with altered renal handling or impaired reabsorption (Law *et al*., 2017; López-Hernández *et al*., 2019; Piras *et al*., 2022).

Experimental and placental studies provide mechanistic context for these systemic observations. In a rat model of GDM, decreased maternal serum 5-HT and increased kynurenine levels were accompanied by altered fetal brain expression of key enzymes, including increased IDO2 and reduced TPH2, indicating a shift toward kynurenine pathway dominance (Zhao *et al*., 2022a). In human placentas from GDM pregnancies, reduced expression and function of 5-HT pathway components, including SERT and the 5-HT_2A_ receptor, have been reported, together with altered DNA methylation of the *SLC6A4* promoter, correlating with maternal glycemic status and neonatal outcomes (Viau *et al*., 2009; Li *et al*., 2014; Blazevic *et al*., 2017). Mechanistically, impaired insulin signaling in GDM may disrupt SERT glycosylation and membrane trafficking, thereby limiting placental 5-HT uptake and availability.

Similarly, gestational hypertension is associated with early disruptions in tryptophan metabolism. Elevated maternal kynurenine concentrations in the first trimester have been linked to impaired uteroplacental vascular development, while increased 5-HTP levels predict early-onset hypertension and heightened preeclampsia risk, suggesting their potential as early biomarkers (van Zundert *et al*., 2024b). Later in pregnancy, women with pregnancy-induced hypertension exhibit elevated plasma tryptophan levels (Grafka *et al*., 2018), while umbilical cord tryptophan concentrations remain unchanged, suggesting a predominantly maternal metabolic dysregulation (Valensise *et al*., 1988).

Given the antioxidant, vasodilatory, and immunomodulatory properties of melatonin, kynurenine, and related indoleamine metabolites, dysregulation of these pathways, particularly during early placental development, may contribute to vascular maladaptation and disease progression. Accordingly, components of the placental tryptophan pathway have emerged as potential biomarkers and therapeutic targets for cardiometabolic complications of pregnancy (Wang *et al*., 2010; Zardoya-Laguardia *et al*., 2018; Broekhuizen *et al*., 2020; Nagasawa *et al*., 2021; Worton *et al*., 2021).

### Early pregnancy complications

Disruptions in placental tryptophan metabolism, particularly within the kynurenine pathway, have been increasingly implicated in early pregnancy complications, including recurrent miscarriage and miscarriage. A consistent finding across clinical and experimental studies is reduced IDO expression and activity at the maternal–fetal interface in affected pregnancies (Broekhuizen *et al*., 2021; Silvano *et al*., 2021). Impaired IDO induction has been observed in dendritic cells and monocytes following CTLA-4 or IFN-γ stimulation (Miwa *et al*., 2005), and reduced IDO expression has been reported in decidual and placental tissues from miscarriages (Ban *et al*., 2013). This downregulation is accompanied by disrupted coordination with Foxp3^+^ regulatory T cells, indicating compromised immune tolerance during implantation and early placentation (Wei *et al*., 2020). Interestingly, IDO expression was higher in chorionic villi from miscarriages with normal karyotypes than in those with chromosomal abnormalities, suggesting the possibility of a potential (albeit insufficient) compensatory response to the genetic abnormality (Obayashi *et al*., 2016).

Beyond immunoregulation, IDO plays a direct role in trophoblast function. In women with unexplained recurrent miscarriages, reduced placental IDO expression correlates with diminished STAT3 phosphorylation and lower MMP9 levels, which are key regulators of trophoblast invasion and placental development (Zong *et al*., 2016). *In vitro*, IDO knockdown impairs trophoblast proliferation and migration, whereas IDO overexpression restores these functions via STAT3 activation (Zong *et al*., 2016). Supporting these findings, recent clinical data demonstrate elevated plasma tryptophan but reduced kynurenine levels in recurrent miscarriage, alongside downregulation of IDO1 in villous and decidual tissues (Jin *et al*., 2025). This was accompanied by downregulation of IDO1 in villous and decidual tissues. Importantly, supplementation with tryptophan or kynurenine improved trophoblast migration, invasion, and angiogenesis *in vitro*, highlighting the functional relevance of the IDO–kynurenine axis in early placental development (Jin *et al*., 2025).

Metabolomic studies further support an immunometabolic imbalance in early pregnancy loss. Broad amino acid profiling in recurrent pregnancy loss revealed elevated tryptophan and 5-HTP levels, accompanied by cytokine dysregulation, suggesting impaired integration of metabolic and immune signals at the maternal–fetal interface (Ye *et al*., 2024). Notably, 5-HTP emerged as a potential diagnostic biomarker. Despite these functional and metabolic associations, common polymorphisms in IDO and TPH genes have not shown strong links to recurrent miscarriage (Unfried *et al*., 2001; Amani *et al*., 2011), indicating that regulatory and environmental mechanisms may outweigh inherited genetic variation.

Recent work has also highlighted the importance of monoamine degradation via MAO during early pregnancy. Adequate MAO activity within the endometrium is essential for decidualization and implantation, and reduced MAO activity leads to accumulation of monoamines such as 5-HT, dopamine, and norepinephrine (Yu *et al*., 2024). This excess monoaminergic signaling disrupts key pathways involved in endometrial receptivity, including aberrant AKT activation, FOXO1 suppression, and impaired decidual gene expression, thereby increasing the risk of implantation failure and early pregnancy loss (Yu *et al*., 2024).

Finally, melatonin has long been implicated in miscarriage risk. Early clinical observations linked low maternal melatonin levels to miscarriage (Sandyk *et al*., 1992), and subsequent studies demonstrated that melatonin supports progesterone synthesis, immune tolerance, antioxidant defense, and uterine quiescence (Chuffa *et al*., 2019). Insufficient melatonin during early pregnancy may therefore weaken multiple protective mechanisms essential for successful implantation and placental establishment (Sandyk *et al*., 1992). Together, these findings emphasize that tight regulation of placental tryptophan metabolism across the kynurenine and 5-HT/melatonin axes is essential for immune tolerance, trophoblast function, and decidual receptivity during early pregnancy. Disruption of these interconnected pathways likely contributes to a substantial proportion of early pregnancy complications (Fig. 3).

### Preeclampsia

Preeclampsia is a multisystem pregnancy disorder characterized by new-onset hypertension and organ dysfunction, driven by placental maladaptation, inflammation, and oxidative stress (Burton *et al*., 2019). Dysregulation of placental tryptophan metabolism, particularly within the kynurenine and 5-HT/melatonin pathways, has been increasingly implicated in its pathogenesis (Fig. 3).

Experimental and clinical evidence support the contribution of altered placental kynurenine pathway regulation to the development of preeclampsia, characterized by divergent regulation of its two entry enzymes, IDO and TDO. In hemi-allogeneic pregnant mouse models, pharmacological inhibition or genetic disruption of IDO recapitulates key features of preeclampsia, including hypertension, proteinuria, and impaired placental perfusion (Nishizawa *et al*., 2008; Santillan *et al*., 2015). Although the onset of preeclampsia does not appear to be driven by primary alterations in *IDO* gene expression, these findings indicate that reduced placental IDO activity contributes functionally to disease development (Nishizawa *et al*., 2010). Accordingly, placentas from preeclamptic pregnancies show reduced IDO expression and activity, with particularly pronounced reductions in late-onset disease (Iwahashi *et al*., 2017; Zardoya-Laguardia *et al*., 2018). IDO expression is also significantly negatively correlated with the severity of maternal hypertension and proteinuria (Iwahashi *et al*., 2017). Notably, this reduction occurs despite the pro-inflammatory milieu characteristic of preeclampsia (Freeman *et al*., 2004). Fresh placental villous explants from affected pregnancies exhibit diminished basal IDO activity and a markedly blunted induction in response to IFN-γ (Kudo *et al*., 2003), indicating impaired inflammatory responsiveness. In contrast, placental *TDO* expression is increased in preeclampsia (Keaton *et al*., 2019), highlighting opposing regulation of the two kynurenine pathway entry points. This pattern suggests either compensatory metabolic reprogramming or fundamentally distinct roles for IDO and TDO in preeclamptic placental pathology, consistent with their differing regulation, tissue distribution, and activation mechanisms (Ball *et al*., 2014). Clarifying how this imbalance shapes downstream kynurenine metabolite profiles may provide critical insight into the vascular dysfunction and immune dysregulation underlying preeclampsia and help identify novel therapeutic targets to restore metabolic homeostasis.

It is worth noting that the published data on tryptophan levels in placental tissue during preeclampsia are inconsistent, likely reflecting heterogeneity in disease subtype and timing. While one study observed significantly reduced placental tryptophan levels in late-onset preeclampsia (Keaton *et al*., 2019), others reported increased placental tryptophan concentrations in early-onset disease (Broekhuizen *et al*., 2019, 2020). These differences suggest gestational stage-specific alterations in tryptophan flux and downstream pathway engagement. Although tryptophan degradation occurs mainly via the kynurenine pathway, the reduction in tryptophan levels seen in late-onset preeclampsia may stem from heightened 5-HT production. Studies have demonstrated elevated placental 5-HT levels in preeclamptic pregnancies, with concentrations correlating positively with disease severity (Poulson *et al*., 1960; Fahim and Botros, 1964). Maternal and fetal serotonergic imbalance in preeclampsia is further supported by increased 5-HT concentrations in umbilical cord plasma (Taniguchi *et al*., 1994), maternal serum (Bhattacharyya and Debnath, 1995), and platelet-poor plasma (Middelkoop *et al*., 1993). Elevated maternal urinary excretion of 5-HT metabolites has also been reported (Filshie *et al*., 1992; Ishii *et al*., 1993). Given the frequent association of preeclampsia with thrombocytopenia (Thiagarajah *et al*., 1984), platelet activation and disintegration likely contribute substantially to increased circulating free 5-HT. Importantly, placental capacity to inactivate 5-HT also appears compromised, as MAO activity is reduced in preeclamptic placentas compared with normotensive controls (Gujrati *et al*., 1996). Because MAO is sensitive to oxygen tension and susceptible to inactivation by reactive oxygen species, placental hypoxia and oxidative stress may also contribute to this loss of function. Increased 5-HT availability, combined with reduced placental degradation, indicates an increased serotonergic burden in preeclampsia, which may contribute to abnormal vascular tone and placental perfusion.

Preeclampsia is also associated with impaired melatonin biosynthesis and signaling. Multiple studies report significantly reduced maternal circulating melatonin levels in preeclamptic pregnancies compared with healthy controls (Nakamura *et al*., 2001; Bouchlariotou *et al*., 2014; Zeng *et al*., 2016; Savka *et al*., 2023). This reduction is accompanied by diminished placental expression of melatonin receptors (MT1 and MT2) and the melatonin-synthesizing enzymes AANAT and ASMT (Lanoix *et al*., 2012; Zeng *et al*., 2016), indicating a broad disruption of placental melatonin signaling. Given melatonin’s potent antioxidative and vasomodulatory properties, these changes may contribute to the oxidative stress and vascular dysfunction characteristic of preeclampsia. Supporting this concept, experimental studies in prenatal and postnatal models demonstrate that exogenous melatonin exerts antioxidant and neuroprotective effects (Welin *et al*., 2007; Miller *et al*., 2014; Aridas *et al*., 2021), prompting clinical evaluations of melatonin as a treatment for preeclampsia and peripartum hypoxia-ischemia (Langston-Cox *et al*., 2021; Fantasia *et al*., 2022). A Phase I clinical trial confirmed maternal and fetal safety and demonstrated a modest prolongation of pregnancy following preeclampsia diagnosis, alongside reduced need for escalation of antihypertensive therapy, although effects on other disease markers were limited (Hobson *et al*., 2018). Importantly, melatonin appears safe even at high doses in pregnancy, and ongoing trials are assessing its therapeutic potential in high-risk pregnancies (Fantasia *et al*., 2022).

### Fetal growth restriction

FGR, characterized by failure of the fetus to achieve its genetically determined growth potential, is strongly associated with placental dysfunction and altered nutrient and oxygen transfer (Laurin *et al*., 1987; Arroyo and Winn, 2008). Tryptophan transport across the placental plasma membranes via system L transporters is substantially reduced in cases of FGR (Lager and Powell, 2012), but despite this, placental tissues from pregnancies affected by FGR reportedly have elevated 5-HT levels (Ranzil *et al*., 2019). This is likely due to increased activity of TPH, the rate-limiting enzyme in 5-HT synthesis. Additionally, both gene- and protein-level expression of SERT are reduced in FGR placentas (Ranzil *et al*., 2019). Enhanced local synthesis combined with impaired 5-HT uptake and clearance is therefore likely to promote placental 5-HT accumulation, potentially disrupting placental signaling and contributing to altered fetal brain development associated with FGR.

In contrast to 5-HT, maternal and umbilical cord melatonin concentrations are significantly reduced in FGR pregnancies (Berbets *et al*., 2020; Berbets *et al*., 2021a), accompanied by downregulation of placental melatonin receptors MT1 and MT2 (Berbets *et al*., 2021b). Functional relevance of this pathway is supported by experimental studies using *Mtnr1b* (MT2) knockout models, which show increased susceptibility to FGR (Wang *et al*., 2024). Therapeutically, maternal melatonin administration alleviates oxidative stress, improves white matter myelination, and enhances neonatal recovery at birth in animal models of FGR (Miller *et al*., 2014; Malhotra *et al*., 2024). In a pilot human trial of FGR pregnancies, melatonin was well tolerated and significantly reduced placental oxidative stress without adverse maternal or fetal effects (Miller *et al*., 2014). Recent data also point to melatonin’s role in mediating fetal growth via the gut–placenta–fetus axis, positioning it as a promising candidate for protecting at-risk pregnancies in the face of rising environmental stressors (Wu *et al*., 2025).

Alterations in the kynurenine pathway further confirm tryptophan dysregulation in FGR. Levels of IDO gene and protein expression (Murthi *et al*., 2017b) as well as functional enzymatic activity (Zardoya-Laguardia *et al*., 2018) were all found to be lower in the placentas of FGR pregnancies than in gestational age-matched controls. Moreover, in experimental models, FGR is also associated with elevated levels of the downstream metabolite QUIN (Sano *et al*., 2016), suggesting impaired flux through NAD^+^-generating branches of the kynurenine pathway. Together, these findings indicate that FGR is characterized by coordinated disturbances across 5-HT, melatonin, and kynurenine pathways, reinforcing the central role of placental tryptophan metabolism in fetal growth regulation.

### Preterm birth

Preterm birth, defined as delivery before 37 weeks of gestation, is commonly associated with intrauterine inflammation and infection, which profoundly disrupt placental structure and function (Hsiao and Patterson, 2011). These inflammatory conditions directly affect placental tryptophan metabolism, with increasing evidence implicating neuroactive metabolites from both the kynurenine and 5-HT/melatonin pathways in adverse fetal outcomes (Bonnin *et al*., 2007; Sedlmayr *et al*., 2014).

Early work by Manuelpillai *et al*. (2005) demonstrated that placentas exposed to infection-associated preterm birth show marked upregulation of kynurenine pathway enzymes, leading to increased production of the neurotoxic metabolite QUIN and elevated QUIN concentrations in umbilical cord blood. Extending these observations, we analyzed a large clinical cohort and identified distinct placental subtypes of preterm birth characterized by differences in gestational age, inflammatory cytokine profiles, and expression of tryptophan pathway genes (Karahoda *et al*., 2021). In a subsequent metabolomic study, we demonstrated altered placental tryptophan flux along the kynurenine pathway in spontaneous preterm birth, with reduced placental kynurenine and KYNA levels alongside elevated QUIN concentrations compared with term controls (Cifkova *et al*., 2024). This metabolic signature mirrors earlier findings in cord blood from preterm infants (Manuelpillai *et al*., 2005) and suggests a shift toward a more neurotoxic kynurenine profile in inflammation-driven preterm placentas.

Alterations in placental kynurenine metabolism that favor QUIN accumulation are of particular concern given the established association between elevated QUIN/KYNA ratios and neuronal injury, mortality, and long-term cognitive impairment in other neurological contexts (Sinz *et al*., 1998; Bell *et al*., 1999; Cogo *et al*., 2021). These findings raise the possibility that placental QUIN dysregulation contributes to the increased risk of neurodevelopmental disorders observed in individuals born preterm (Saigal and Doyle, 2008). Future studies should therefore integrate placental metabolic profiling with long-term neurodevelopmental follow-up, accounting for gestational age, inflammatory burden, and placental tryptophan pathway activity. Ongoing work in our laboratory aims to directly link placental QUIN levels with neurodevelopmental outcomes in preterm-born children.

In parallel, preterm birth is associated with profound disruption of melatonin physiology. Preterm infants exhibit markedly lower circulating melatonin levels than term infants (Merchant *et al*., 2013; Biran *et al*., 2019). This can be attributed to both the sudden loss of maternal melatonin at birth and the pronounced immaturity of fetal melatonin synthesis in preterm infants, which causes endogenous production to be delayed by 2–3 weeks compared to term infants (Kennaway *et al*., 1992). This extended period of melatonin deprivation renders the preterm brain particularly vulnerable to perinatal brain injury (Jan *et al*., 2007).

Experimental studies support a protective role for melatonin in this context. In animal models of inflammation-induced preterm birth, maternal melatonin administration attenuated inflammatory responses, reduced rates of preterm delivery, and mitigated offspring brain injury (Domínguez Rubio *et al*., 2014; Lee *et al*., 2019). Early clinical studies further indicate that melatonin supplementation in preterm infants can restore circulating melatonin to physiological levels. Intravenous melatonin administration achieved measurable plasma concentrations, although slow clearance complicated circadian rhythm mimicry (Merchant *et al*., 2013). More recently, oral melatonin supplementation in very preterm infants increased circulating melatonin and 6-hydroxymelatonin levels and was associated with reduced lipid peroxidation, suggesting a potential antioxidant benefit (Garofoli *et al*., 2024). Together, these findings indicate that preterm birth is characterized by coordinated disruptions in placental kynurenine metabolism and neonatal melatonin availability, both of which may contribute to the adverse neurodevelopmental outcomes often associated with preterm birth. While melatonin replacement emerges as a promising adjunctive strategy, further controlled clinical trials are required to optimize dosing, timing, and long-term efficacy in preterm populations.

## Investigative approaches and models for studying placental tryptophan dynamics

Direct investigation of placental tryptophan metabolism *in vivo* in humans is constrained by ethical and technical limitations, necessitating the use of complementary experimental models. Animal studies have been pivotal in defining how maternal diet, stress, inflammation, and hypoxia shape placental function and fetal exposure to neuroactive metabolites (Fando *et al*., 1981; Badawy, 1988; Arevalo *et al*., 1991; Huether *et al*., 1992; Tsuji *et al*., 2013; Notarangelo and Schwarcz, 2016; Sano *et al*., 2016; Goeden *et al*., 2017; Williams *et al*., 2017; Moura *et al*., 2022). Rodent and sheep models are particularly valuable for mechanistic work because they allow controlled manipulation of maternal and placental environments that is not feasible in humans. *In situ* placental perfusion models in rodents replicate key features of placental transport and metabolism and have provided important insight into 5-HT and tryptophan handling at the maternal–fetal interface (Bonnin *et al*., 2011; Karahoda *et al*., 2020b; Horackova *et al*., 2021). Additionally, our studies on pregnant sheep have shown that impaired placental function increases placental levels of QUIN, a neurotoxic kynurenine metabolite (Nicholls *et al*., 2001a,b; Yan *et al*., 2005).

Despite these strengths, translation is limited by interspecies differences in placental architecture, gestational timing, parturition, and regulation of tryptophan-metabolizing enzymes, and by the fact that major human disorders such as preeclampsia do not occur spontaneously in most species (Badawy, 2015; Marshall *et al*., 2018). Within these constraints, the Wistar rat is considered among the most suitable rodent models for placental tryptophan metabolism due to its metabolic characteristics and placental gene-expression profile (Badawy, 2015; Racicot and Latham, 2017). This assessment is supported by our own comparative analyses, which demonstrate substantial overlap between rat and human placental expression of key transporters and enzymes involved in tryptophan metabolism (Abad *et al*., 2020). Emerging evidence in species such as the cortisol-secreting spiny mouse (*Acomys dimidiatus*) may further enhance translational relevance owing to their more human-like hemochorial placentation (O’Connell *et al*., 2013).

*In vitro* placental models offer greater experimental control but vary widely in physiological relevance. Choriocarcinoma-derived cell lines such as BeWo (including the BeWo-b30 clone) and JEG-3 are frequently used but exhibit major limitations for tryptophan research, including undetectable expression of key genes such as *IDO1* and *OCT3* (Entrican *et al*., 2002; Karahoda *et al*., 2020a), with OCT3 deficiency confirmed at the functional level (Vachalova *et al*., 2022). In contrast, primary human trophoblast cells closely mirror term placental tissue in the expression of tryptophan transporters and metabolic enzymes (Karahoda *et al*., 2020a; Baković *et al*., 2021; Vachalova *et al*., 2022) and therefore represent the most physiologically relevant *in vitro* system, despite limitations in availability and lifespan (Rosario *et al*., 2013; Aye *et al*., 2015; Gaccioli *et al*., 2015; Ferchaud-Roucher *et al*., 2017; Silva *et al*., 2024). Additionally, trophoblast organoids represent a major methodological advance, recapitulating key features of early human placental development and enabling differentiation into syncytiotrophoblast and extravillous trophoblast lineages (Turco *et al*., 2018). Although not yet applied to studies of tryptophan metabolism, their cellular complexity and relevance to early gestation make them promising platforms for investigating regulatory mechanisms of the 5-HT/melatonin and kynurenine pathways under defined conditions.

To overcome the limitations of isolated cell systems and capture later gestational physiology, several *ex vivo* models based on intact human placental tissue have been developed. The *ex vivo* perfused human term placenta remains a gold-standard model for studying nutrient and drug transport, as well as placental metabolism (Myllynen *et al*., 2005; Hutson *et al*., 2011; Stirrat *et al*., 2018). This approach has been successfully applied to investigate placental handling of tryptophan (Broekhuizen *et al*., 2020) and 5-HT (Mathiesen *et al*., 2020; Staud *et al*., 2023). Given the placenta’s role in regulating fetal exposure to 5-HT, this model may offer key insights into how disrupted tryptophan metabolism might affect fetal brain development.

Complementary approaches include isolated microvillous and basal membrane vesicles (Illsley *et al*., 1990), which enable high-throughput assessment of tryptophan and 5-HT transporter-mediated processes on individual placental membranes (Ganapathy *et al*., 1986; Balkovetz *et al*., 1989; Kudo and Boyd, 2001; Karahoda *et al*., 2020b; Horackova *et al*., 2021). However, the absence of regulatory and multicellular interactions limits extrapolation to *in vivo* physiology. Villous fragments (Greenwood and Sibley, 2006) and placental explants (Kudo and Boyd, 2001) provide an intermediate level of complexity, preserving tissue architecture and cellular interactions while remaining experimentally tractable. These models, particularly placental villous explants, have been instrumental in studying placental tryptophan, 5-HT, and kynurenine transport and metabolism under physiological and pathological conditions, including inflammation and FGR (Kudo and Boyd, 2001; Ligam *et al*., 2005; Manuelpillai *et al*., 2005; Murthi *et al*., 2017b; Abad *et al*., 2024; Staud *et al*., 2023).

Taken together, currently available placental models offer complementary perspectives on tryptophan metabolism across gestation and disease contexts. Careful selection of experimental systems, matched to the biological question, developmental stage, and pathway of interest, is essential for generating physiologically meaningful and translatable insights into placental tryptophan dynamics.

## Future directions

Placental tryptophan metabolism is now recognized as a tightly regulated network that links maternal supply and placental transport to enzymatic conversion along the 5-HT/melatonin and kynurenine pathways. Nevertheless, important questions remain about how this regulation is established across gestation and how early shifts relate to pregnancy and offspring outcomes. A central priority is early pregnancy, when placental perfusion and metabolic capacity are still developing, and even modest changes in transporter activity or pathway engagement may alter fetal exposure to neuroactive metabolites before fetal tissues can regulate these systems autonomously. In particular, defining how early pregnancy conditions influence the downstream kynurenine balance, including the KYNA/QUIN ratio, which reflects the relative availability of neuroprotective versus potentially neurotoxic metabolites, will be important for understanding fetal vulnerability and later neurodevelopmental outcomes. Addressing these questions will require mechanistic studies that combine human-relevant placental models (e.g. primary trophoblasts, explants, organoids, and *ex vivo* perfusion where feasible) with carefully selected animal approaches that allow control of timing and tissue sampling, while explicitly accounting for gestational stage and species differences.

In parallel, clinical research needs to move to early pregnancy and become more hypothesis-driven. Prospective cohorts should begin in the first trimester and include longitudinal collection of maternal biospecimens and pregnancies at high risk (obesity, GDM, chronic inflammation, and hypertensive disorders), where pathway dysregulation may be detectable before clinical presentation. Beyond describing circulating profiles, studies should aim to link maternal metabolite patterns to placental expression and activity where possible, and then to pregnancy endpoints and postnatal follow-up. Nutritional and pharmacological interventions, including vitamin D and melatonin, remain promising. However, their mechanisms, optimal timing, and placental targets need to be defined to determine whether shifting pathway balance in early pregnancy influences placental function, fetal development, and pregnancy outcomes. Finally, maternal nutrition in low- and middle-income settings warrants greater attention, as variation in dietary tryptophan availability and micronutrient status may influence placental metabolite output and downstream developmental risk. This will require prospective, interdisciplinary studies that integrate nutrition, placental biology, and developmental follow-up.

## Conclusions

This review highlights the placenta as a central regulator of tryptophan metabolism at the maternal–fetal interface, with tightly controlled, gestation-dependent regulation. Rather than being static, placental tryptophan metabolism adapts to changing physiological demands across pregnancy and responds to maternal and environmental influences such as inflammation, oxidative stress, and cardiometabolic dysfunction. Disruption of this regulatory network is associated with a broad spectrum of pregnancy complications, ranging from early pregnancy loss to later gestational disorders, including preeclampsia, FGR, and preterm birth, highlighting the knowledge that placental control of tryptophan metabolism is integral to normal placental function and pregnancy maintenance. Importantly, these alterations also have direct consequences for fetal neurodevelopment, as both 5-HT- and kynurenine-derived metabolites contribute to key processes beginning in early gestation, including neurogenesis, neuronal migration, axonal growth, synapse formation, and myelination (Goeden *et al*., 2016; Beecher and Wixey, 2023). During early gestation, placenta-derived 5-HT plays a critical role in the development of the forebrain in the fetus, whereas excessive serotonergic signaling in response to maternal inflammation can disrupt axonal patterning and influence the development of brain circuits (Bonnin *et al*., 2011; Goeden *et al*., 2016). As gestation advances, 5-HT synthesis shifts toward the fetal brain, but it is now clear that early disturbances of tryptophan catabolism in the placenta have long-lasting effects. In parallel, maternal immune activation and inflammatory exposures shift placental tryptophan metabolism toward the kynurenine pathway, altering the balance between neuroprotective and potentially neurotoxic metabolites (Williams *et al*., 2017; Abad *et al*., 2024). In this context, the KYNA/QUIN balance emerges as a key indicator of fetal neurodevelopmental vulnerability.

Emerging evidence further links placental tryptophan metabolism to fetal neuroendocrine regulation. 5-HT influences adrenal development (Kameneva *et al*., 2022), while glucocorticoids (both physiologically normal and stress-induced) can redirect kynurenine pathway flux toward specific neuroactive outcomes (Jovanovic *et al*., 2023). These interactions point to a complex interplay between placental tryptophan metabolism, the hypothalamic–pituitary–adrenal axis, and brain development, which begins *in utero* and may extend into postnatal and adult life. This concept aligns with the DOHaD framework, suggesting that early disturbances in placental function and fetal neuroimmune-endocrine signaling shape the vulnerability of the postnatal brain to neuropsychiatric and metabolic disorders. Thus, placental tryptophan metabolism acts both as a mediator of fetal programming and a potential point of clinical intervention. In parallel, melatonin has gained attention as a promising therapeutic candidate because of its antioxidant, vasomodulatory, and neuroprotective properties. Taken together, these findings support the concept that placental tryptophan metabolism is both a key mediator of fetal programming and a modifiable target for interventions aimed at improving pregnancy outcomes and long-term child health.

## Data Availability

No new data were generated or analyzed in support of this research.

## Abbreviations list

3-HAA: 3-hydroxyanthranilic acid
3-HAAO: 3-hydroxyanthranilate 3,4-dioxygenase
3-HK: 3-hydroxykynurenine
5-HIAA: 5-hydroxyindoleacetic acid
5-HT: serotonin (5-hydroxytryptamine)
5-HTP: 5-hydroxytryptophan
AANAT: aralkylamine *N*-acetyltransferase
ASMT: *N*-acetylserotonin O-methyltransferase
DDC: DOPA decarboxylase
DOHaD: developmental origins of health and disease
DOPA: dihydroxyphenylalanine
EAAT: excitatory amino acid transporter
GDM: gestational diabetes mellitus
IDO: indoleamine 2,3-dioxygenase
IFN-γ: interferon gamma
IL-6: interleukin-6
FGR: intrauterine growth restriction
KAT: kynurenine aminotransferase
KMO: kynurenine monooxygenase
KYNA: kynurenic acid
LAT: system L amino acid transporter
LPS: lipopolysaccharide
MAO: monoamine oxidase
MRP2: multidrug resistance-associated protein 2
MT: melatonin receptor
NEFA: non-esterified fatty acids
NMDA: *N*-methyl-D-aspartate
OAT: organic anion transporter
OCT: organic cation transporter
QUIN: quinolinic acid
SERT: serotonin transporter
TDO: tryptophan 2,3-dioxygenase
TNF: tumor necrosis factor
TPH: tryptophan hydroxylase
